# Response of soil water content temporal stability to stand age of *Haloxylon ammodendron* plantation in Alxa Desert, China

**DOI:** 10.3389/fpls.2023.1099217

**Published:** 2023-01-24

**Authors:** Dongmeng Zhou, Jianhua Si, Xiaohui He, Bing Jia, Chunyan Zhao, Chunlin Wang, Jie Qin, Xinglin Zhu, Zijin Liu

**Affiliations:** ^1^ Key Laboratory of Eco-Hydrology of Inland River Basin, Northwest Institute of Eco-Environment and Resources, Chinese Academy of Sciences, Lanzhou, China; ^2^ University of Chinese Academy of Sciences, Beijing, China; ^3^ Faculty of Resources and Environment, Baotou Teachers’ College, Inner Mongolia University of Science and Technology, Baotou, China

**Keywords:** soil water content, *Haloxylon ammodendron* plantations, temporal stability, spatiotemporal variability, stand age

## Abstract

Afforestation as an effective measure for wind and sand control has achieved remarkable results in northern China, and has also greatly changed the land use and vegetation characteristics of the region. It is important to study the spatial and temporal dynamics of soil water content (SWC) in different afforestation years and its temporal stability to understand the dynamic characteristics of SWC during afforestation. In order to reveal the spatiotemporal dynamic characteristics of SWC in desert area *Haloxylon ammodendron (HA)*plantations, in this study, five restorative-aged *HA* plantations in desert areas were selected and their SWC was measured in stratified layers for the 0–400 cm soil profile; we also analyzed the spatiotemporal dynamics and temporal stability of the SWC. The results showed that the SWC of *HA* plantations decreased with the increase in planting age in the measurement period, and the SWC of deep layers increased by more than that of shallow layers with planting age. Spearman’s rank correlation coefficients for SWC of 0–400 cm in both 5- and 11-year-old HA plantations reached above 0.8 and were highly significantly correlated; the temporal stability of SWC tends to increase as the depth of the soil layer deepens. In contrast, the temporal stability of SWC in deeper layers (200–400 cm) of 22-, 34- and 46-year-old stands showed a decreasing trend with depth. Based on the relative difference analysis, representative sampling points can be selected to monitor the regional average SWC, but for older *HA* plantations, the uncertainty factor of stand age should be considered in the regional moisture simulation. This study verified that it is feasible to simulate large-scale SWC in fewer observations for *HA* plantations younger than 11 years old, while large errors exist for older stands, especially for deeper soils. This will help soil moisture management in *HA* plantations in arid desert areas.

## 1 Introduction

This study area is located in northwestern China, at the southern edge of the Badain Jaran Desert, which has long suffered from wind and sand. In order to effectively curb wind and sand hazards and prevent the further expansion of sandy land, China has launched a number of major ecological construction projects in wind and sand hazards, such as the “3-North Shelter Forest Program” and the “Grain for Green” program with artificial vegetation construction as the main ecological restoration measure. The vegetation represented by *Haloxylon ammodendron* (HA) plantations is increasingly restricted by soil water content (SWC), especially as the large area of *HA* plantations has a more obvious soil drying phenomenon, which seriously affects the water cycle process in the study area (Kang et al., 2021; Zhou et al., 2022). Therefore, it is important to understand the hydrological effect of SWC after afforestation for the water cycle and eco-hydrological process of terrestrial ecosystems. SWC is at the core of functioning sandy ecosystems in arid and semi-arid regions, driving the material cycle and energy flow in the soil–vegetation–atmosphere continuum, and its dynamic changes affect hydrological and ecological processes such as precipitation infiltration, vegetation transpiration and solute transport (Vereecken et al., 2015; Zhang et al., 2016). SWC has strong spatial and temporal variability due to topography, elevation, soil texture and climate (Hu et al., 2010; Heathman et al., 2012). Related studies also indicate that vegetation affects the spatial distribution of SWC, enhancing or reducing the spatial heterogeneity of SWC to some extent (Li et al., 2008; Cho and Choi, 2014), and the variability of SWC also responds to vegetation growth succession and spatial patterns to varying degrees (Van Pelt and Wierenga, 2001). Therefore, a quantitative study of the spatial variability of SWC is essential to grasp the regional ecohydrological dynamics.

Although soil water has strong spatial and temporal variability, previous studies have shown that SWC is characterized by temporal stability (Brocca et al., 2009; Brocca et al., 2010). Vachaud et al. (1985) found that the spatial structure of SWC is continuous in time when external factors such as soil structure and topography remain stable, and that certain sampling points can represent regional average SWC conditions; this phenomenon was subsequently defined as the temporal stability of SWC. Since then, the temporal stability of SWC has been widely used to identify the average SWC condition in the field (Huang et al., 2020; Zhou et al., 2020; Quan et al., 2021) to simulate hydrological variables (Nasta et al., 2018). The temporal stability of SWC is influenced by numerous elements, such as the length of the observation period (Zhang et al., 2018) and the type of land use (Liu and Shao, 2014; Zhang et al., 2016). Liu and Shao (Liu and Shao, 2014) reported a significant effect of different land use types on SWC in the 0–4 m profile and further validated the feasibility of representative sampling points for estimating the average SWC. Zhang et al. (Zhang et al., 2016) reported that in the spring wheat–shelterbelt–maize agroforestry ecosystem, SWC relationships between adjacent land use types were explored by determining the most time-stable locations of each soil profile for different land use types. Jia and Shao (Jia and Shao, 2013) reported that vegetation cover and above-ground biomass are the main factors affecting the temporal stability of SWC, and further concluded that sampling in slopes may produce better results when the temporal stability theory is applied to slopes.

The above findings indicate that the study of temporal stability of SWC is more meaningful in the context of non-homogeneous soils, and that vegetation factors profoundly affect temporal stability and differ for different soil depths. As a key area for revegetation in the Alxa desert region, artificial revegetation has greatly changed the land use and vegetation characteristics of the region, and the effect of this huge disturbance on the temporal variability and temporal stability of SWC is yet to be studied. Specifically, this study aimed to: (1) explore the response of SWC to stand age in HA plantations;(2) reveal the spatiotemporal dynamic characteristics of SWC in desert area. This study provide a theoretical basis for water resource management and vegetation construction in the region.

## 2 Materials and methods

### 2.1 Description of study site

The Alxa desert area is located in northwestern China (97°10′E-106°52′E,37°21′N-42°47′N). The region has an arid climate. The sunshine is sufficient and the annual sunshine hours are 2993 to 3345h. The annual average temperature is 6.8-8.8°, with extreme minimum and maximum temperatures of -36.4° and 41.7° respectively. The temperature has a large diurnal temperature difference and significant seasonal changes. The frost-free period is 130-165 d. The average annual precipitation is 39.3-85.6 mm, with precipitation from July to September accounting for about 90% of the year. The water table is 80-120 m, and there are no river confluences. Due to the drought tolerance and high survival rate of HA, the Chinese government planted HA in large quantities in the study area around the 1970s for wind and sand control, and it has played a vital role in the improvement of the ecological environment in the area.

### 2.2 Experimental design and measurements

Since 1975, afforestation projects have been carried out every year in Alxa desert. In this study, the HA planted in 1975,1987,1999,2010 and 2016 were selected according to the principle of consistency of soil texture. Then, five representative sample plots of 5-, 11-, 22-, 34- and 46-year-olds were selected in the study area with a 10-year age gradient, 50m*50m sample plots were delineated in each plantation and 3 replicate samples were collected at the center of the sample plots in May–September 2021 in 20 cm stratification. The 0-400 cm soil profile was sampled in 20 cm layers, and three duplicate samples were collected from each sample site. And put the soil sample into the aluminum box back to the laboratory for drying method to measure the SWC. Three undisturbed soil samples were collected with a ring knife near the sampling point for the determination of soil hydraulic characteristics such as soil bulk density and field water capacity, and sample plots were investigated at the same time (**Table 1**). The selected plots were all planted in a standardized “two rows and one strip “planting mode. There was no other vegetation around, so the study was not interfered with by the planting density and other vegetation on the experimental results.

**Table 1:** Characteristics of *Haloxylon ammodendron* (HA) plantation lands at different plots.

Parameters of sites	Plot 1	Plot 2	Plot 3	Plot 4	Plot 5	Plot 6
Planting Age (a)	5	11	22	34	46	Wasteland
Year of planting	2016	2010	1999	1987	1975	/
Altitude (m)	1204.86	1204.95	1203.69	1203.66	1204.37	1203.97
Clay Volume Fraction (%)	11.07	11.61	11.73	11.16	10.97	10.31
Silt Volume Fraction (%)	13.57	15.95	14.26	13.19	12.80	15.18
Sand Volume Fraction (%)	75.36	72.44	74.01	75.65	76.23	74.51
Diameter at breast height(cm)	3.5 ± 1.2	8.9 ± 3.8	12.4 ± 5.5	16.5 ± 6.2	22.1 ± 7.5	/
Mean tree height/(cm)	55 ± 12	140 ± 28	220 ± 46	370 ± 63	420 ± 81	/
Tree density/(Tree/hm^2^)	230	230	230	230	230	/
Bulk Density/(g/cm^3^)	1.53	1.53	1.52	1.53	1.54	1.53

### 2.3 Statistical analysis

Soil water storage is the amount of soil water stored in a certain unit volume. In this study, based on the observed depth of 0–400 cm soil depth, the soil water storage per unit volume of 0–400 cm soil depth is given by:


(1) $${\text{SWS}}_{i}=\sum_{i=1}^{20}{\text{SVWC}}_{i}d_{i}$$

(2) $$\bar{{\text{SVWC}}_{j}}=\frac{1}{20}\sum_{i=1}^{20}{\text{SVWC}}_{ij}$$

where SWS_*i*_ is a soil water storage of 0–400 cm from point i (i=1,…,n) (mm), SVWC_*i*_ is the soil volumetric water content (cm3cm-3), *d*_*i*_ is the soil depth (mm), and $\bar{{\text{SVWC}}_{j}}$ is the average soil volumetric water content at time j (j=1,…,m) (cm3cm-3), The number of soil layers observed in this study is 20. According to Vachaud et al. (1985), the relative difference (RD) and standard deviation (SD) of each observation can characterize the temporal stability of SWC. The *RD*_*ij*_ and *SD* of SWS_*i*j_ at any observation at point i (i=1,…,m) at time j (j=1,…,m) are given by:


(3) $$RD_{ij}=\frac{{\text{SVWC}}_{ij}-\bar{{\text{SVWC}}_{j}}}{\bar{{\text{SVWC}}_{j}}}$$

(4) $$SD=\sum_{i=1}^{m}\sqrt{\frac{{\text{SVWC}}_{ij}-\bar{{\text{SVWC}}_{j}}}{m-1}}$$

where SVWC_*ij*_ is SVWC at point i (i=1,…,m) at time j (j=1,…,m) (cm3cm-3), $\bar{{\text{SVWC}}_{j}}$ is the average SVWC at time j (j=1,…,m) (cm3cm-3), and m is the number of measurements. The mean relative difference MRD_*i*_ and its corresponding standard deviation SDRD_*i*_ are given by:


(5) $${\text{MRD}}_{i}=\frac{1}{m}\sum_{j=1}^{m}RD_{ij}$$

(6) $${\text{SDRD}}_{i}=\sqrt{\frac{1}{m-1}\sum_{i=1}^{m}{\left(RD_{ij}-{\text{MRD}}_{i}\right)}^{2}}$$

According to Zhao et al. (Zhao et al., 2010), we compared the temporal stability of SWC at different soil depths in different restoration years of HA plantations by comparing the index of temporal stability at depth i (ITSD_*i*_ ) to find the highest temporal stability point. The observation point with the highest temporal stability, which is representative of the average SWC condition and ITSD_*i*_ , is given by:


(7) $${\text{ITSD}}_{i}=\sqrt{{\text{MRD}}_{i}^{2}+{\text{SDRD}}_{i}^{2}}$$

**Figure 1:**
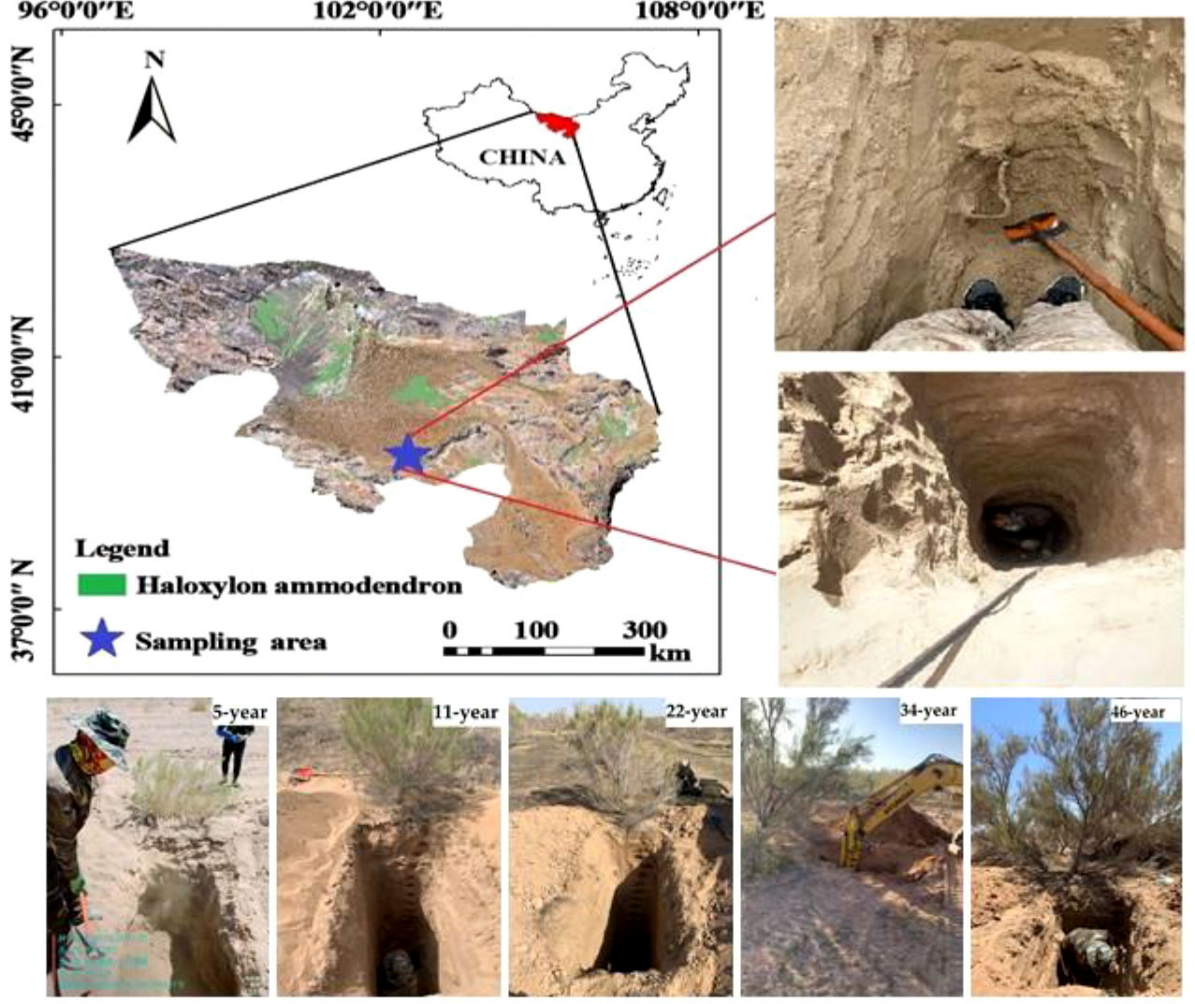
Location of the study area.

Spearman’s rank correlation coefficient *r*_*s*_ was used to analyze the stability of the rank change over time for different observations during the growing season:


(8) $$r_{s}=1-6\sum_{i=1}^{n}\frac{{\left(R_{ij}-R_{il}\right)}^{2}}{n\left(n^{2}-1\right)}$$

where *R*_*ij*_ is the rank of the SWC at point i at time j,*R*_*il*_ is the rank of the SWC at point i at time l, and n is the total number of observation points. Dummy **Figure 1**

## 3 Results and discussion

### 3.1 Distribution characteristics of SWC variability

**Figure 2:**
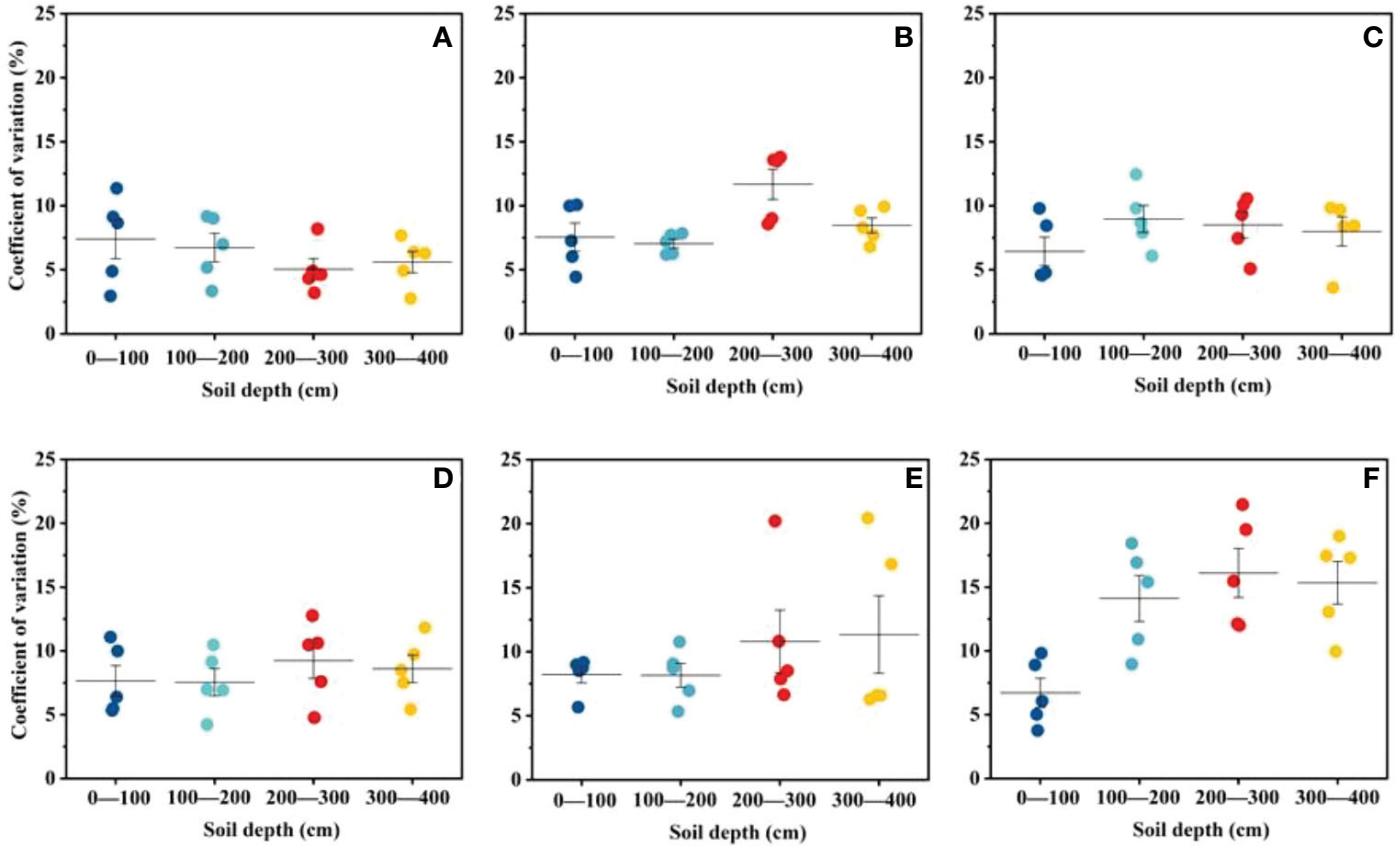
A scatterplot of SWC coefficient of variability for each soil layer in HA plantation forested land at different ages: **(A)** wasteland, **(B)** 5, **(C)** 11, **(D)** 22, **(E)** 34 and **(F)** 46 years. Different colors represent different soil depths: blue (0-100 cm), green (100-200 cm), red (200-300 cm), yellow (300-400 cm).

Stand age influenced the spatial and temporal distribution of soil water to some extent, and that the age factor gradually overshadowed the soil depth factor as the stand age increased. **Table 2** shows the spatial statistical characteristics of the mean SWC, standard deviation and coefficient of variation (CV) of the 0–400 cm soil layer profile in each HA plantation. The standard deviation and CV of SWC in each soil layer showed a general trend of increasing with the deepening of the soil layer. From **Figure 2**, it can be seen that the CV of SWC changed irregularly for 5-,11-, and 22- year-old of HA plantations, but the CV of SWC of 34- and 46-year-old HA plantations tends to increase with the deepening of the soil layer, It is indicated that the spatial variability of SWC increases with the deepening of the soil layer for 34- and 46-year-old of HA plantations. Li et al. (2015) reached similar conclusions on the Loess Plateau, the spatial distribution of SWC depends largely on structural factors such as climate, topography and soil texture, while stochastic factors such as vegetation recovery and human activity increase the spatial variability of SWC (Zhao et al., 2018). The sampling area is located at the southern edge of the Badain Jaran Desert, and is less disturbed by human and animal activities, the spatial variability of SWC was increase as the soil layer deepened for 34- and 46-year-old HA plantations. Nan et al. (2019) quantified the soil water depletion of artificial *Robinia pseudoacacia* forest of different ages (5, 20, and 40 years) on the Loess Plateau. His research showed that soil water consumption was divided into two stages, the first stage was that the artificial *Robinia pseudoacacia* forest gradually transited from shallow soil layer to deep soil layer with the increase of age, the second stage is the transition from the deep soil to the shallow soil when the deep soil water consumption reaches a certain threshold. However, this study found that the soil water consumption was similar to the first stage of the above research with the increase of the age of the HA plantation, but the rule of the second stage did not appear. The reason for this difference may be that the sampling depth of this study is relatively shallow (400 cm), and the sampling depth has not yet reached the conversion threshold of soil water consumption. According to Zeng et al. (2011), it was found that the root system of aged HA can grow vertically down to 800 cm, which also verified the above hypothesis. Future studies should carry out deeper soil layer research for aging HA. It also helps to reveal the relationship between artificial vegetation growth and soil water consumption.

**Table 2:** Spatial statistical characteristics of temporal average, standard deviation and coefficient of variation (CV) of SWC in different soil layers.

Planting Age (a)	Variable	Soil depth (cm)			
		0–100 cm	100–200 cm	200–300 cm	300–400 cm
5	Mean SWC	7.56	6.34	7.29	8.41
	Standard deviation	0.59	0.45	0.85	0.71
	CV	7.56	7.04	11.68	8.47
11	Mean SWC	7.65	6.20	6.04	7.40
	Standard deviation	0.48	0.37	0.50	0.58
	CV	6.43	5.94	8.49	7.99
22	Mean SWC	7.48	5.95	5.36	6.40
	Standard deviation	0.57	0.45	0.49	0.54
	CV	7.65	7.54	9.24	8.60
34	Mean SWC	6.71	5.36	4.13	4.65
	Standard deviation	0.55	0.44	0.45	0.52
	CV	8.21	8.16	10.80	11.35
46	Mean SWC	6.33	2.87	2.42	2.36
	Standard deviation	0.42	0.39	0.39	0.36
	CV	6.71	14.11	16.10	15.34

### 3.2 The spatiotemporal behavior of SWC

**Figure 3:**
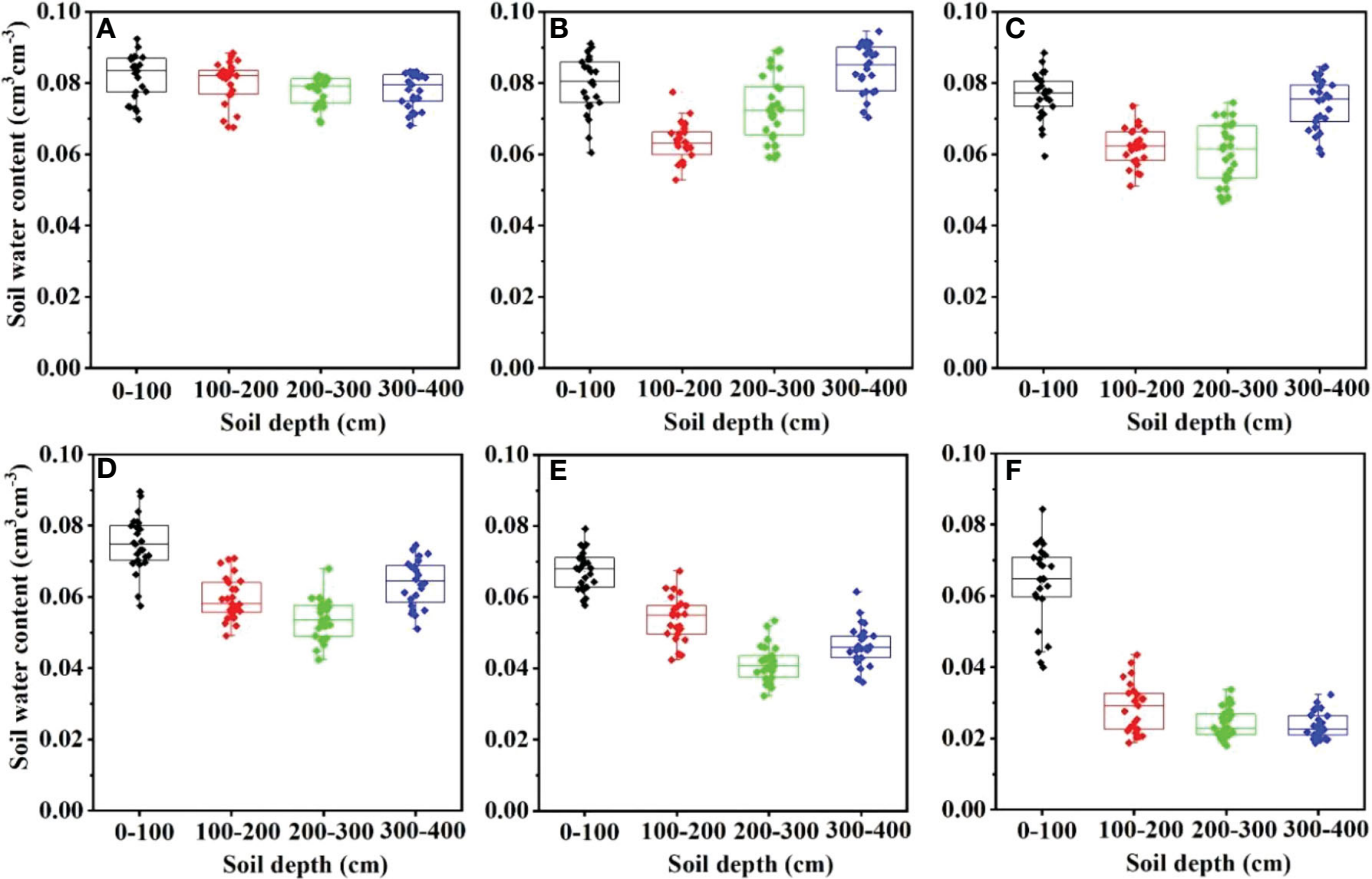
A boxplot of the vertical distribution of soil water content (SWC) in HA plantations: **(A)** wasteland, **(B)** 5-year-old, **(C)** 11-year-old, **(D)** 22-year-old, **(E)** 34-year-old and **(F)** 46-year-old.

The SWC showed a trend of decreasing with increasing years of HA plantations, and the variation of SWC with plant years was greater in the deep layer than in the shallow layer. **Figure 3** shows the variation trend of SWC with depth in the HA plantation profile at each stand age; it can be seen that the SWC in the surface layer (0–100 cm) did not vary much with stand age during the study period and was at a high level relative to the deeper layers, fluctuating in the range of 0.06-0.09 cm^3^cm^-3^. The SWC in the 100–400 cm soil layer varied greatly with the age of the forest, fluctuating between 0.02-0.09 cm^3^cm^-3^, with the SWC in the 100–400 cm soil layer decreasing with depth in the 46-year-old HA plantations. The SWC in 11-, 22- and 34-year-old HA plantations showed a decreasing trend with a depth from 0 to 300 cm, but there was an “inflection point” from 300 to 400 cm, where the SWC increased with depth and the “inflection point” in 5-year-old HA plantations occurred at a more shallow level (100–200 cm). Hu et al. (2010) used soil water storage data in the Loess Plateau to identify temporal stability points, and found that artificial vegetation significantly affected deep soil water. Gao and Shao (2012) conducted research on 0-300 cm soil layer SWC in the Loess Plateau and found that the change rate of SWC in deep soil was lower than that in shallow soil, which was contrary to the conclusion of this study. These differences are due to the different types of trees, shrubs and herbs that were focused on in the study of Gao and Shao (2012). The present study is consistent with Hu et al. (2010), which was conducted on a mono-vegetated hillside. Compared to trees and shrubs, herbs consume less water at shallow depths, and the effect of vegetation factors on SWC varies with soil depth. The vegetation factors interfered with the regularity of SWC variation with depth, resulting in large differences in vertical soil profile variations under different vegetation types. In addition, this study area is located in an arid desert area with little human activity, which reduces the influence of human activity and other factors on the soil surface moisture changes. HA plantations are planted for a long period of time (46 years) and HA is a deep-rooted plant (Gong et al., 2019); the SWC in the 200–400 cm soil layer is more influenced by root water consumption, which also leads to the deep SWC changing with planting. The SWC in the 200–400 cm soil layer is more influenced by root water consumption, which leads to a greater variation of SWC in the deep layer than in the shallow layer with planting age.

**Figure 3** shows that there is no significant decrease in SWC in the 0–100 cm soil profile for all stand ages, while the SWC in 200–400 cm decreases significantly after more than 11 years of HA plantations. Moradi et al. (2017) assessed the impact of afforestation on soil physical and chemical properties and SWC in south-west Iran, and found that afforestation improved soil conditions but significantly depleted SWC. Haque et al. (1999) studied differences in SWC in three different plantations in the North-East of Scotland. The results showed that, compared with the control group, the early growth stage had little effect on SWC, while the late growth stage made the soil dry. Wang et al. (2021) reached similar conclusion in HA plantations of different planting years in the desert oasis transition zone of the Hexi Corridor. Guo et al. (2016), however, found that the SWC in HA plantations significantly increased in the 260 cm soil layer. The main reason for the difference is that Guo et al. (2016) selected HA plantations which were 3 years old; compared with the short planting period in this study, the root depth of HA plantations in 3-year-old stands is shallow. In comparison, Wang et al. (2021) selected HA planting years similar to the present study. The reason for this phenomenon is that precipitation is low and evaporation is high in the study area. The less precipitation mainly occurs in short precipitation periods, and the infiltration process of precipitation is mainly concentrated in the soil layer within 60 cm. Therefore, shallow soils can be recharged by precipitation, while deep soil water recharge is not significant. (Yang and Zhao, 2014). Furthermore, the transpiration water consumption of HA is much higher than the precipitation (Chang et al., 2007); after 22 years of planting in particular, the excessive consumption of deep soil water by HA roots further leads to SWC deterioration.

### 3.3 Temporal stability of SWC

#### 3.3.1 Spearman’s rank correlation coefficient

**Figure 4:**
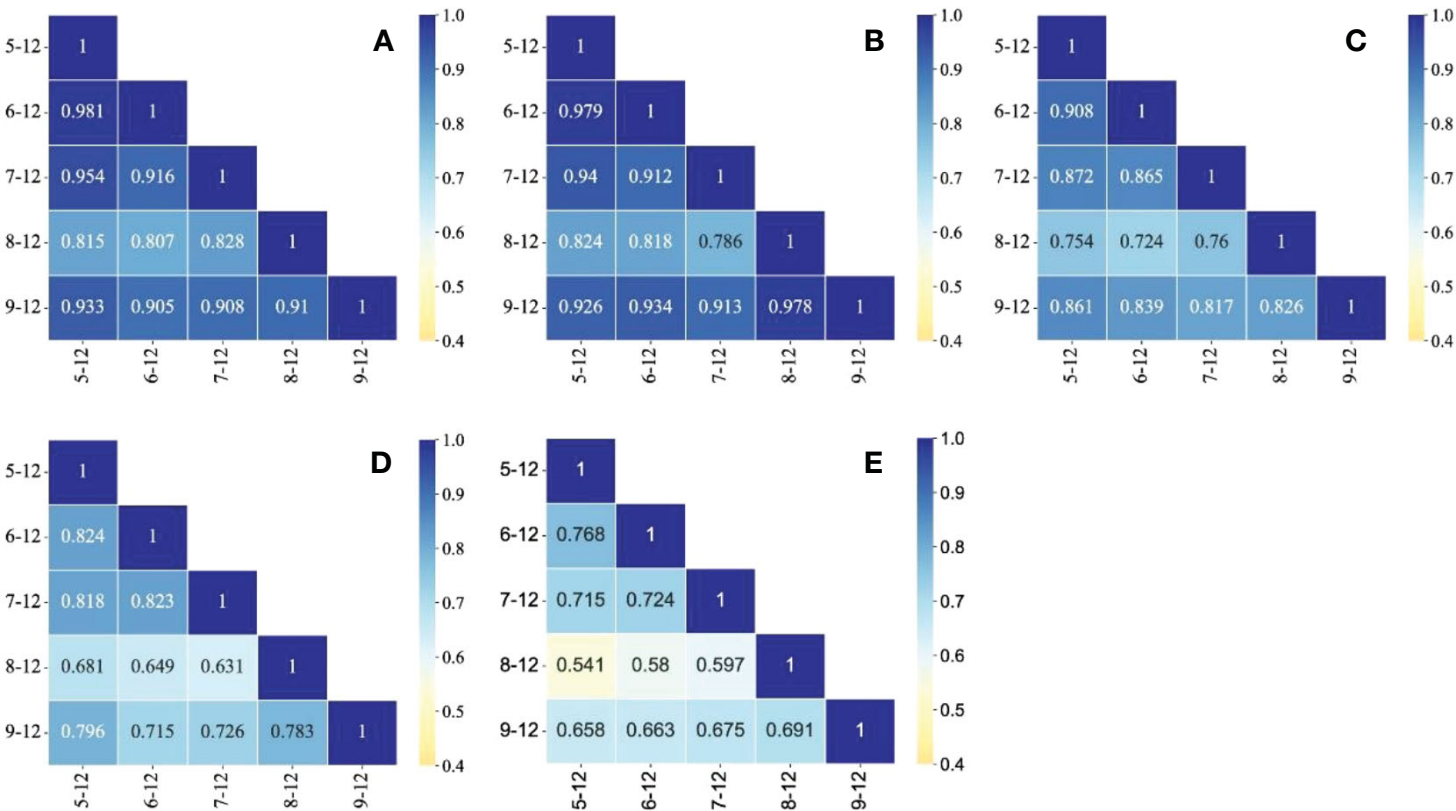
Spearman’s rank correlation coefficient heat map of 0-400 cm SWC in *HA* plantations: **(A)** 5-year-old, **(B)** 11-year-old, **(C)** 22-year-old, **(D)** 34-year-old and **(E)** 46-year-old.

For different stand ages, the correlation coefficients tended to decrease with increasing stand age, but in general the correlation coefficients were greater than 0.5 and highly significant (P<0.01). Spearman’s rank correlation coefficient of SWC at each sampling point can characterize the temporal stability of the study area as a whole. **Figure 4** shows Spearman’s rank correlation coefficient matrix of SWC at 0–400 cm depth for different observation dates in the study area. As depicted in **Table 2**, the correlation coefficient between August 12 and the other measurement dates is small, between 0.631 and 0.828, and the correlation coefficient between the remaining dates is large. The reason for this discrepancy may be due to the large variation in SWC due to the larger rainfall in the week before the July 12 sampling, resulting in a smaller correlation coefficient. The study of Xin et al. (Xin et al., 2008) on the temporal stability of soil water uptake also found that the temporal stability of soil water was worst when the soil was alternately dry and wet. This indicates that the spatial distribution of SWC at 0–400 cm depth in *HA* plantations in the study area is characterized by temporal stability within the study time; Grant et al. (2004), However, it should be noted that the temporal stability showed a trend of time-dependent variation, The closer the sampling time, the greater the correlation coefficient. This result indicates that the duration of temporal stability of SWC is limited, which is a similar conclusion in other studies with interannual observation scales (Penna et al., 2013; Zhang and Shao, 2013a). This study was conducted based on one growing season of *HA* plantations, and for future studies, a longer time series can be considered to explore the effective period of temporal stability.

#### 3.3.2 Relative difference analysis

**Figure 5:**
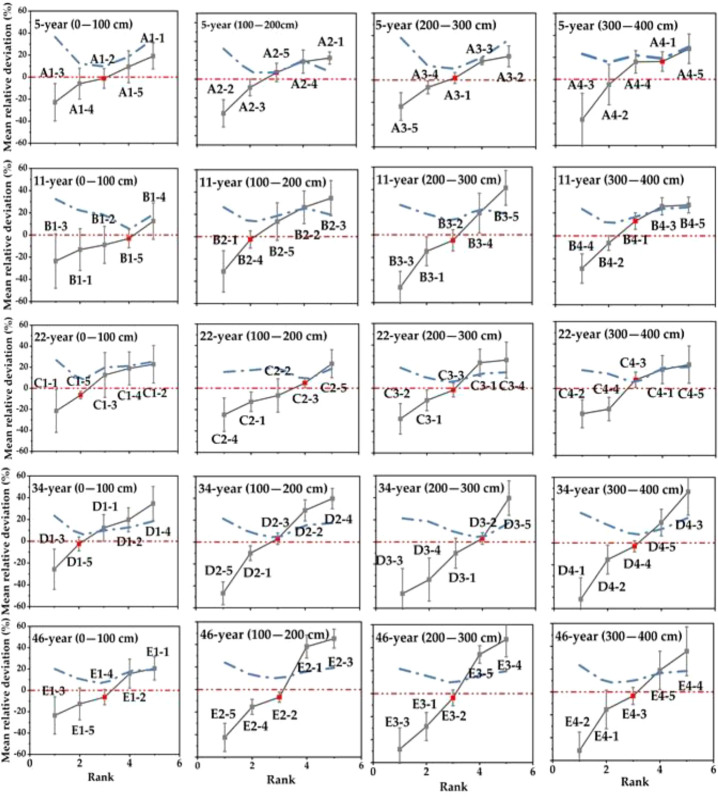
Ranked mean relative difference (MRD) of SWC in the diverse soil layers in HA plantations, the letters in the figure represent different stand ages: **(A)** 5-year-old, **(B)** 11-year-old, **(C)** 22-year-old, **(D)** 34-year-old and **(E)** 46-year-old. The numbers in the figure are the sample point numbers. The solid gray line indicates MRD, the red line is the baseline with an average relative deviation of 0. Error bars represent the standard deviation of relative difference (SDRD). The most time-stable locations are indicated by the red dots. The blue dotted line indicates index of temporal stability at depth.

Deep SWC in favor of maintaining a higher temporal stability. However, with the introduction of artificial vegetation, the growth of stand age gradually overshadows the soil depth factor, making the temporal stability of deep SWC lower. **Figure 5** shows the mean relative difference (MRD) values of SWC at each sampling point in different soil layers in descending order, and the corresponding time index of temporal stability at depth curves; the vertical error line is the standard deviation of relative difference (SDRD) at each sampling point. The MRD of SWC of 5- and 11-year-old *HA* plantations showed a trend of increasing and then decreasing; for the shallow layer, the MRD increased gradually probably because the spatial variability of SWC increased as the soil layer deepened (Jia and Shao, 2013). The MRD of SWC in the deeper soil layers decreases, probably due to the young age of the *HA* plantations and the failure of the root system to grow into the deeper soil layers. For the 22-, 34- and 46-year-old *HA* plantations, the MRD in SWC increased with the increasing depth of the soil layer, and the same conclusion was reached in the CV analysis above. The stronger the spatial variability, the more dispersed the distribution of water content at each sampling point, and the greater the deviation from the average value will be; Zhang and Shao (2013) selected two plots with different soil textures in northwest China, and evaluated the spatial distribution of SWC. The results showed that the deeper the soil depth, the greater the MRD of SWC, which was similar to the conclusion of this study. In a gravel–mulch field in northwestern China, in relation to the spatial and temporal stability of SWC, Zhao et al. (Zhao et al., 2017) found that the MRD decreases with increasing soil depth; the reason for this difference lies in its small research scope (32*32 m), and the gravel–mulch field soil has a homogeneous texture and uniform sand and gravel cover, resulting in its weak spatial variability of SWC. Li et al. (Li et al., 2015) found that the fluctuation range of the MRD may be related to the study scale, test placement, sampling method, etc. With the expansion of the study area, the more complex the corresponding soil properties, topography and vegetation cover, the stronger the spatial variability of SWC, and the range of the MRD also increases (Martinez-Fernandez et al., 2003). Spatial variability is the result of the interaction of different dominant factors at different spatial scales, and in the process of scale transformation, the influencing factors also change, so the results of studies at different scales vary (Sun et al., 2022). Therefore, for specific research objects, the sampling scale range and sampling granularity need to be considered in order to have a better explanation of the spatial heterogeneity of SWC (Fagan et al., 2003). **Figure 4** also shows that the MRD is asymmetric, with negative absolute values greater than positive values, due to the fact that the SWC at more sampling points is less than the mean value, probably because the soil at the measurement site has a gravel content, which is not conducive to water retention.

The magnitude of the SDRD can characterize the degree of temporal stability of SWC at the sampling point; the smaller the SDRD, the higher the temporal stability. **Figure 4** shows that 5-,11- and 22-year-old HA plantations is in line with the trend of decreasing SDRD with soil depth deepening, but not at ages 34 and 46, indicating that deep soil water of HA plantations at the age of 5,11, and 22 is not disturbed by vegetation and has higher temporal stability. However, SWC in the deep layer of HA at 34-,46-year-old was greatly disturbed by vegetation, so the temporal stability of SWC did not show a decreasing trend with soil depth., which was consistent with the MRD analysis above. Temporal stability of SWC is the result of the interaction of many factors such as vegetation, topography, climate and soil (He et al., 2021). The study area is on the edge of the desert. The climate environment of low rainfall and high evaporation has its own special effects on the temporal stability of soil water; long time soil freezing makes the mid-soil flow a predominantly vertical movement; meanwhile, the soil dry layer induced by vegetation water depletion hinders the vertical movement of soil water and limits the recharge of deep soil water. Ding et al. (Ding et al., 2020) concluded that artificially restored vegetation has a certain impact on SWC in the rhizosphere, but it has less impact below the rhizosphere, mainly because the vegetation growth period is short and the vegetation characteristics of slow growth make its ecological water consumption low, and the precipitation basically meets the vegetation growth demand. However, this study found that as the growth years of *HA* plantations increased, it had a non-negligible effect on the changes of SWC dynamics; precipitation and evaporation are the most direct influencing factors of SWC, but there are large differences in response to different soil depths. Surface runoff caused by strong rainfall on the slope surface replenishes surface SWC significantly; while it is not conducive to deep SWC infiltration and evaporative dissipation of surface SWC is large, the rough soil texture is weak in lifting deep SWC, and the combined factors lead to deep SWC in favor of maintaining a higher temporal stability. However, with the introduction of artificial vegetation, the growth of stand age gradually overshadows the soil depth factor, making the temporal stability of deep SWC lower.

The results of the above-mentioned study showed that the temporal stability of deep soil layers was higher than that of shallow layers, and the temporal stability characteristics of SWC were depth-dependent for *HA* plantations before 22 years of age. This is consistent with the results of related studies (Gao and Shao, 2012a). However, for older *HA* plantations there is an opposite performance in deeper soils (200–400 cm). It is worth to note that, limited by the experimental conditions, this study only studied SWC in HA plantation with a stand age of 5,11,22,34, and 46 years, and could not find a continuous stand that met the experimental conditions. Therefore, appropriate models should be selected for future research in order to evaluate SWC more accurately.

#### 3.3.3 Representative sampling locations

**Figure 6:**
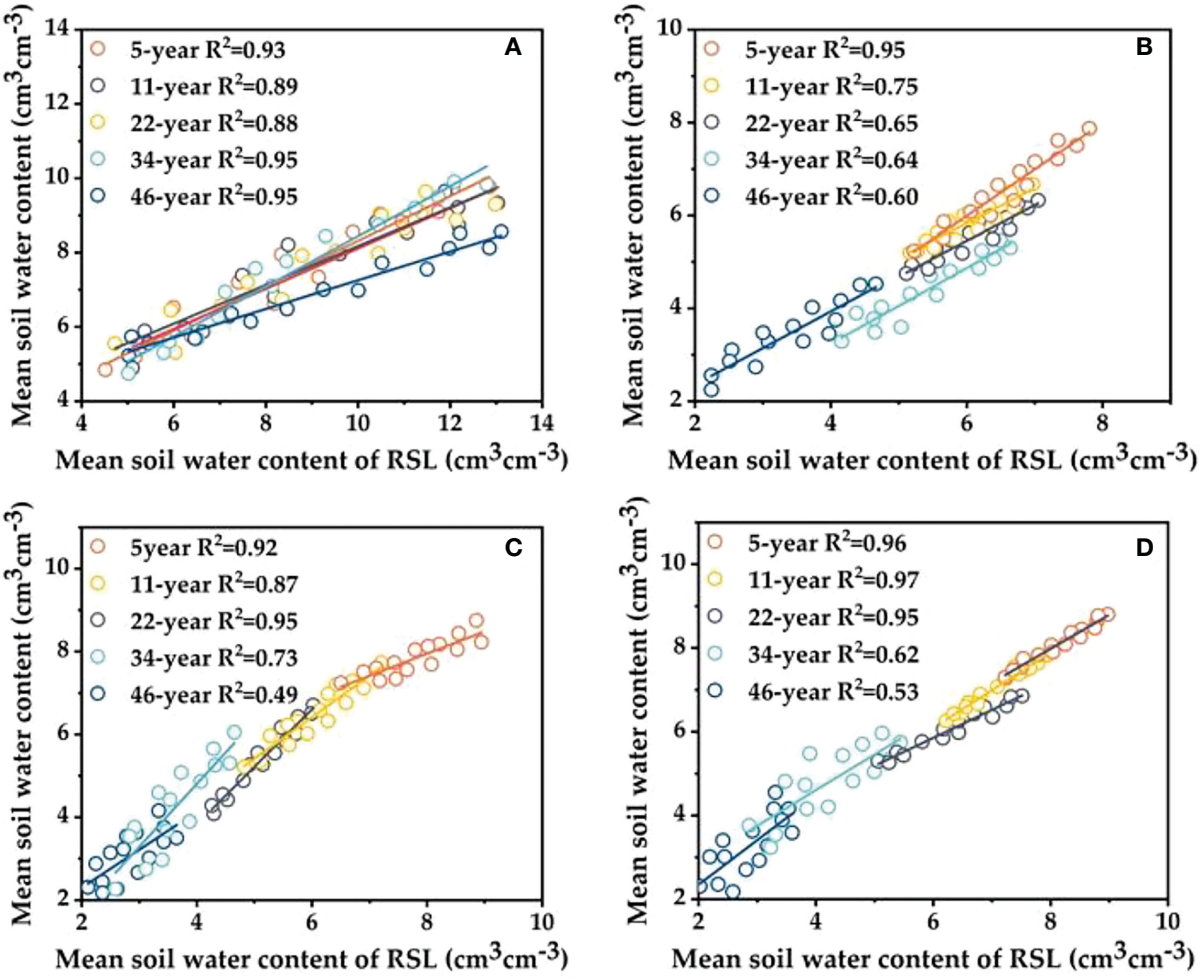
Comparisons of measured SWC at the most time-stable location with the estimated values in the four soil layers under five different stand ages: **(A)** 0—100 cm, **(B)** 100—200 cm, **(C)** 200—300 cm, **(D)** 300—400 cm.

The correlation with the mean value of SWC in the corresponding soil layer is high, and the average SWC of each soil layer in the study area can be estimated more accurately. Zhang et al. (Zhang et al., 2016) show that representative sampling locations (RSL) can be selected to estimate the regional average SWC according to the principle that the MRD is close to 0 and the SDRD is relatively small. To verify the reasonableness of the RSL, the SWC of each RSL was compared with the average SWC of each soil layer, and it was found that the SWC of each measurement point fluctuated slightly around the average SWC (**Figure 6**). Meanwhile, the regression analysis between the mean SWC values of different soil layers and the RSL during the observation period showed that the coefficient of determination R^2^ of each soil layer from 0 to 400 cm varied in the ranges of 0.88–0.95, 0.60–0.95, 0.49–0.95 and 0.53–0.95, indicating that the correlation between the mean SWC values of each RSL and the corresponding soil layer was high. The correlation with the mean value of SWC in the corresponding soil layer is high, and the average SWC of each soil layer in the study area can be estimated more accurately, but the temporal stability of the deep SWC gradually decreases with increasing growth years of *HA* plantations.

### 3.4 Implications for future afforestation activities

In order to prevent further desert expansion, China introduced many plant species in the 1970s (Ahrends et al., 2017). The excessive water consumption of artificial vegetation has broken the dynamic balance between precipitation and native vegetation, and many new environmental problems have emerged (Gao and Shao, 2012b; He et al., 2019). In recent years, there have been numerous studies showing that the massive consumption of soil water by artificial vegetation leads to a decrease in SWC and has a negative impact on vegetation growth, which in turn threatens the health of the ecosystem (Chang et al., 2007; Xin et al., 2008; Zhang and Shao, 2013).

In our field survey, we found that when the SWC dropped to 0.02-0.03 cm^3^cm^-3^ after 30a of *HA* planting, the leaves fell off in large numbers and the individual biomass decreased significantly. Moreover, when the SWC was at 0.01-0.02 cm^3^cm^-3^, the sand-fixing plants appeared to decline; at about 1% the sand-fixing plants died in large numbers. Although *HA* can reduce the water consumption of aging photosynthetic organs by withering leaves and branches, the water scarcity of old leaves, branches and stems is extremely serious and has basically reached its limit. The further shedding of a large number of leaves and branches reduces its individual biomass and deteriorates its survival ability. Finally, the stems also dry up and break due to too little water in the plant. Therefore, based on the above findings, we suggest that moderate manual interventions, such as pruning of dead branches, proper irrigation and some level of crop flattening or intercropping after 30–40a of HA planting, may have positive implications for the revival and rejuvenation of *HA* plantations. It is worth noting that this study based on the same planting density on the SWC of different age HA plantations, to avoid the planting density to interfere with the results of the study. In the actual afforestation project, usually through adjusting the planting density to reduce the deep soil water consumption. Therefore, future research should further establish mechanism models through vegetation growth and soil water consumption to provide theoretical basis for rational afforestation engineering.

## 4 Conclusions

1. The SWC of 0-400 cm of *Haloxylon ammodendron* plantations t in the study area showed a decreasing trend with the increase of HA planting age, and the HA plantations consumed mainly shallow soil water in 5- and 11-year-old stands, while 22-,34-and 46- year-old consumed mainly deep soil water.

2. The distribution of SWC at 0-400 cm depth in the *HA* plantations in the study area was characterized by temporal stability during the study time period, but it should be noted that the temporal stability showed a time-dependent trend, with the correlation coefficient increasing the closer the sampling time and tending to decrease as the time interval increased. This result indicates that the duration of temporal stability of SWC is limited.

3. Afforestation (e.g., *Haloxylon ammodendron*) is an important measure to prevent wind and sand fixation in desert areas, which is of great significance to the sustainable development of the ecosystem. Our study found that the age of vegetation has an important influence on the deep SWC dynamics, and with the increase of forest age, the water depletion of vegetation roots leads to the degradation of SWC in the study area, and the temporal stability also decreases with the increase of forest age and soil depth, therefore, in the future afforestation work in the Alxa desert area, appropriate moisture control measures should be taken for *HA* plantations of advanced forest age.

## Data availability statement

The original contributions presented in the study are included in the article/**Supplementary Material**. Further inquiries can be directed to the corresponding author.

## Author contributions

DZ: conceptualization, data curation, investigation, methodology, formal analysis, roles/writing—original draft, writing—review and editing. JS: conceptualization, data curation, formal analysis, funding acquisition, methodology. BJ: investigation. CZ: conceptualization, data curation, formal analysis, investigation. JQ: investigation, XH: investigation. CW: investigation. XZ: investigation. ZL: investigation. All authors read and approved the final manuscript. All authors contributed to the article and approved the submitted version.
